# Conversion of lignocellulosic waste into effective flocculants: synthesis, characterization, and performance

**DOI:** 10.1186/s40643-021-00422-1

**Published:** 2021-08-03

**Authors:** Elham Jahedi, Reza Panahi

**Affiliations:** Chemistry & Chemical Engineering Research Center of Iran (CCERCI), Tehran, Iran

**Keywords:** Cationization, Cellulose fibers, Flocculation mechanism, Natural flocculant, Rice husk, Turbidity removal

## Abstract

Development of cationic flocculants from lignocellulosic wastes not only eliminates the health and environmental concerns associated with the use of conventional chemicals, but also is the way of waste valorization. In the present study, cellulose fibers extracted from rice husk were cationized through an optimization method based on response surface methodology. The fibers cationized at the optimal conditions had a zeta-potential of 15.2 ± 1.0 mV, while the highest potential was + 8.76 mV, for the samples developed before optimization. FTIR analysis proved the presence of the corresponding functional groups. The functionalized fibers were biodegradable and had absolutely positive surface charges at a broad pH range. The cationized fibers were employed as a flocculant to remove turbidity from the synthetic wastewaters at various pHs and initial turbidities. The cationic fibers showed the excellent turbidity removals up to 98.5% from the synthetic wastewater without the need for conventional coagulants. In contrast to traditionally cationized fibers, the synthesized flocculants did not affect the effluent color during coagulation–flocculation. The charge neutralization and bridging through adsorption were the governing mechanisms of flocculation. The procedure can be applied on lignocellulosic wastes to develop cationic fibers with the excellent flocculation ability and suitable operational characteristics.

**Supplementary Information:**

The online version contains supplementary material available at 10.1186/s40643-021-00422-1.

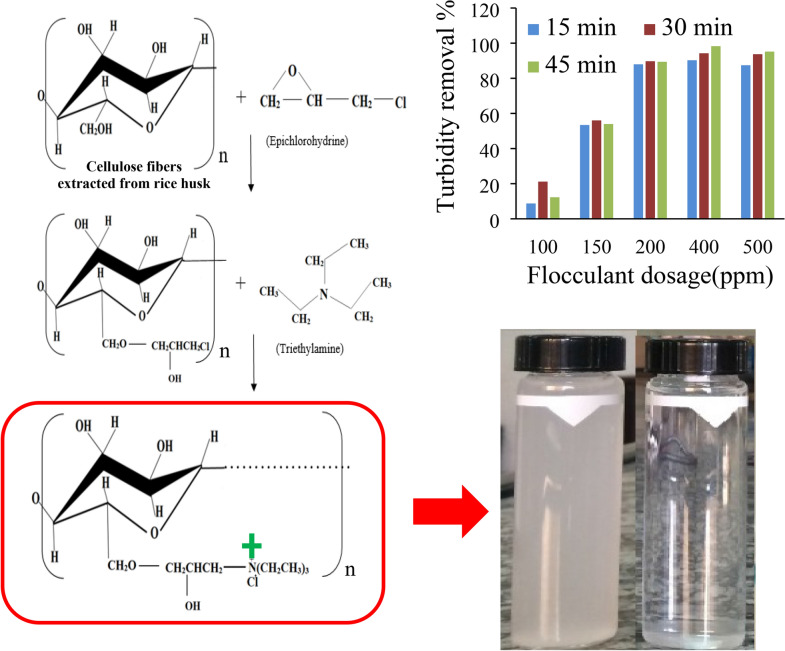

## Introduction

Nowadays, the control of water pollution is a critical global challenge due to insufficiency of freshwater, existence of serious water pollution, and increment of world population. Hence, new methods and technologies being robust and available at low cost, offering a short processing time and leaving minimal impact on the environment particularly those based on biomolecules and biomasses are of interest (Ferasat et al. 2020; Abdollahi et al. 2019, 2018, 2017; Liu et al. 2017; Ebrahimi et al. 2009).

The coagulation–flocculation process is a common part of water and wastewater treatment. This process removes the suspended colloidal particles and various dissolved contaminants from water bodies in cost effective and easy manner (Ferasat et al. 2020; Du et al. 2017; Yang et al. 2016). In coagulation, the repulsive potential of colloids is reduced and micro-particles are formed. Subsequently, these micro-particles aggregate to form larger structures (flocs) in the flocculation step. The larger flocs are effectively removed by sedimentation (Matilainen et al. 2010). The long-established coagulants are mostly Al- or Fe-based. Polyaluminum chloride (PACl), polyaluminum sulfate, polymeric ferric sulfate, and polymeric ferric chloride are examples of cationic coagulants (Tang et al. 2015). Flocculants can be anionic (hydrolyzed polyacrylamide; polyacrylic acid; polyvinyl sulfate), cationic (diallyl dimethylammonium chloride; polyethylene imine; polyvinyl pyridine) or non-ionic (polyethylene oxide; polyacrylamide; polyvinyl alcohol) in nature (Gregory and Barany 2011). The employment of Al-based flocculants results in the presence of aluminum ion in the treated water, which is connected with the appearance of Alzheimer’s disease and dialysis encephalopathy syndrome (Tassinari et al. 2013). Moreover, remaining trace monomers in effluents due to the application of synthetic organic flocculants particularly polyacrylamide or acrylamide leads to potential adverse health effects. Such a monomer is potentially toxic and carcinogenic (Lapointe and Barbeau 2017). On the other hand, the presence of such chemicals in the produced sludge causes the environmental concerns.

The development and application of new cationic flocculants from renewable sources, e.g., agricultural waste can be an impressive action to overcome these challenges. It brings about several advantages such as deleting the need for coagulant, valorizing the waste, being nature friendly, and reducing the cost of wastewater treatment.

Cellulose is known as the most abundant natural polymer in the world, so it has a considerable potential to develop functional materials (Soltani Firooz et al. 2017). Cellulose fibers can be extracted from lignocellulosic wastes such as rice husk. Cationization of cellulose fibers is mainly performed using epichlorohydrin and triethylamine. This reaction involves at least two serious problems. First, considerable amount of catalyst, e.g., pyridine is used in the reaction. Pyridine has recently been classified as a hazardous material on the basis of its low occupational exposure limit value (Prat et al. 2016). Second, such cationized fibers give color to the effluent during wastewater treatment.

In this work, cellulose fibers were extracted from rice husk and used as a backbone to develop a cationic flocculant. To deal with the highlighted challenges, cationization of the fibers was conducted and optimized in the absence of catalyst, which has not been reported before, to the best of our knowledge. The characterization and biodegradability of the modified fibers were investigated. Furthermore, the cationic fibers were employed to remove colloidal particles from kaolin suspensions at various pHs and turbidities. This application of rice husk has rarely been discussed in previous studies.

## Material and methods

### Materials

Raw rice husk was prepared from the north of Iran. Epichlorohydrin, triethylamine, dimethylformamide, sodium chlorite and sodium hydroxide were from Merck. Acetic acid (Fluka, Switzerland) and kaolin (Duksan, Korea) were used in the experiment. All chemicals were analytical grade. Commercial PACl (65% basicity and 31% alumina) was used for comparison. Deionized water was used for all the experiments.

### Preparation of cellulose fibers

Raw rice husk was washed with water in order to eliminate dust and contaminants, afterwards it was dried (at 50 °C for 24 h) and ground. The biomass was screened to reach the particle size lower than 425 µm. Rice husk particles (20 g) were treated with 200 ml NaOH solution (4% w/v) at 80 °C for 2 h under rigorous mixing in order to remove hemicellulose and lignin from the cellulose fibers. This treatment was repeated for 3 times. After each step, the suspension was filtered and washed with excess water. Then, two procedures were individually used for bleaching. The cellulose fibers were bleached using a solution of NaOH and acetic acid in the preliminary experiments (Oliveira et al. 2017). Alternatively, the cellulose fibers were re-suspended in 200 ml aqueous chlorite (1.7 wt. %), followed by adding acetic acid to the fiber suspension, such a way that the color of the suspension changed. The mixture was incubated for 4 h at 100 °C under rigorous mixing. This procedure continued by filtering the suspension and washing the particles with plenty of water. The bleaching was repeated for 4 times (Johar et al. 2012).

### The cationization of cellulose fibers

**Fig. 1:**
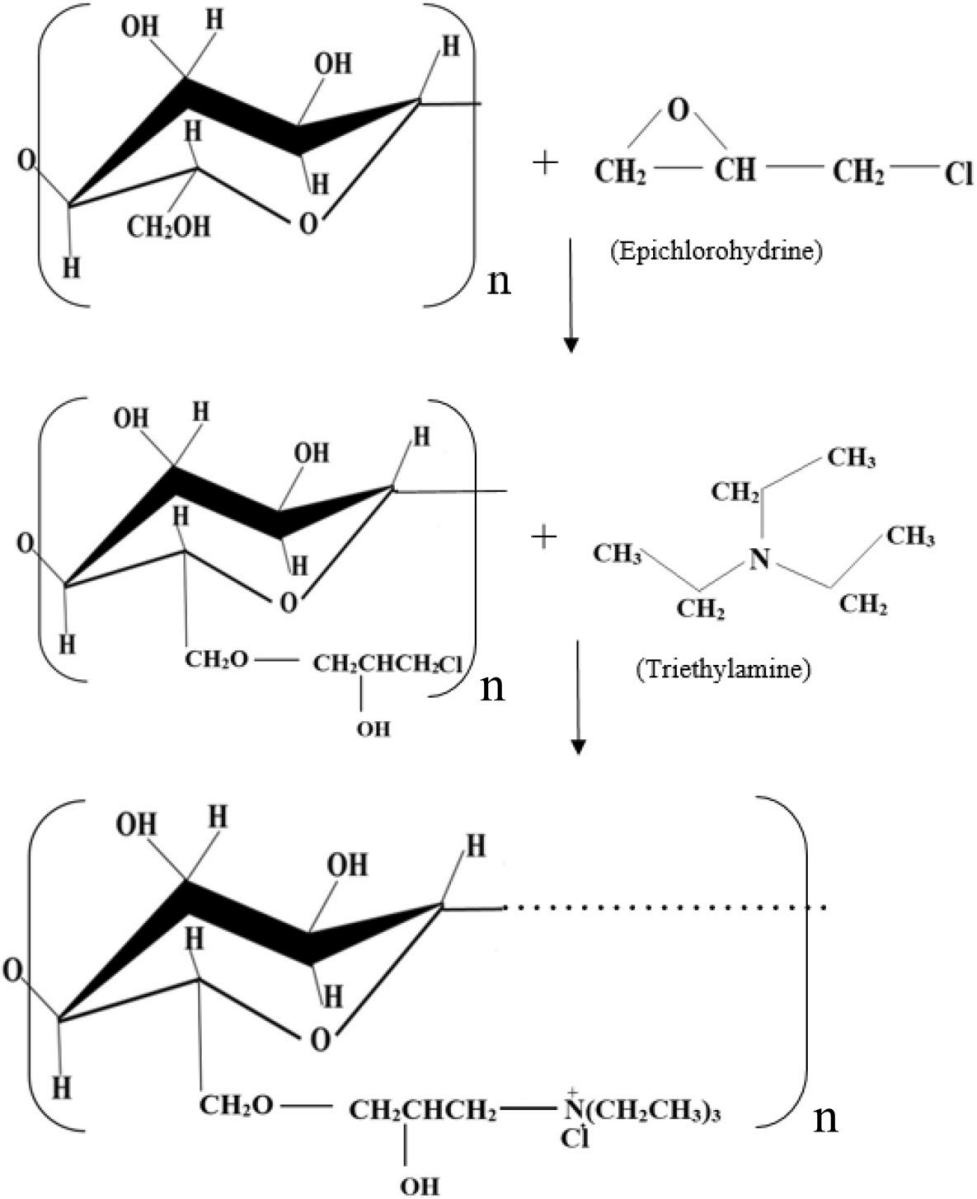
Synthesis reactions for the cationization of cellulose fibers

The cationization of cellulose fibers was performed according to the previous study (Gao et al. 2009) with modification. To this end, the dried cellulose fibers were dispersed in dimethylformamide. Epichlorohydrin was added to the suspension and the reaction was carried out at 100 ºC for 1 h under gentle stirring to functionalize the fibers (Fig. 1). Then, triethylamine was added to the mixture and the reaction followed for 3 h to obtain cationic cellulose fibers. The fibers were separated from the suspension, washed with plenty of water, and dried at 50 °C for 12 h. For the catalytic cationization, pyridine was added to the mixture containing the functionalized fibers, and the suspension was stirred for 1 h at 50–100 °C, before adding triethylamine. Finally, the fibers were ground to achieve uniform particles smaller than 710 µm. The amounts of reactants to synthesize the preliminary samples are reported in Table 1.

**Table 1:** The amount of reactants in the preliminary experiments

Reactants	Run no. (product no.)			
	P1	P2	P3	P4
Cellulose source	Bleached fiber	Bleached fiber	Bleached fiber	Raw husk
Epichlorohydrin (ml)	10	6	2	2
Triethylamine (ml)	20	4	12	12
Cellulose suspension (g fiber/ml DMF)	0.4 g/10 ml	2.0 g/10 ml	1.0 g/10 ml	1.0 g/10 ml

DMF: dimethylformamide

### Experimental design and data analysis

The amounts of cellulose, epichlorohydrin, and triethylamine in the reaction environment were optimized to maximize zeta-potential of the synthesized fibers via response surface methodology (RSM). The coded levels and actual values of the factors are shown in Table 2. The experiments were conducted according to Box–Behnken design. The design uses several center points to provide an estimate of experimental error and to verify the adequacy of the model. In addition, Box–Behnken designs never include runs where all factors are at their extreme setting, e.g., the highest levels. As reported in Table 3, this design suggests a limit number of experiments. A cellulose suspension containing 1 g fibers per 15 ml dimethylformamide was used as a fiber source in all synthesis experiments. The relationship between the three variables and the responses (zeta-potential of the fibers) can be expressed by a second-order polynomial equation. At the optimal values of the variables, several experiments were conducted to validate the accuracy of the optimization. The design of experiments, analysis of variance (ANOVA), and the optimization process were performed using Design-Expert software.

**Table 2:** The coded and actual values of the variables for the synthesis of cationic fibers based on Box–Behnken design

Factors	Levels			Actual values (ml)		
	Low	Mean	High	Low	Mean	High
Epichlorohydrin (A)	− 1	0	+ 1	2	4	6
Triethylamine (B)	− 1	0	+ 1	8	10	12
Cellulose suspension (C)	− 1	0	+ 1	7.5	15	22.5

**Table 3:** Coded levels of factors in the experiments suggested by Box–Behnken design

Run	Coded levels of the factors^a^		
	A	B	C
1	− 1	+ 1	0
2	− 1	0	+ 1
3	0	− 1	− 1
4	+ 1	+ 1	0
5	− 1	− 1	0
6	0	+ 1	− 1
7	+ 1	0	− 1
8	+ 1	− 1	0
9	0	+ 1	+ 1
10	0	0	0
11	0	− 1	+ 1
12	0	0	0
13	− 1	0	− 1
14	0	0	0
15	+ 1	0	+ 1

^a^*A* epichlorohydrin, *B *triethylamine, *C* cellulose suspension

### Characterization

Fourier transform infrared (FTIR) spectra of the chemically functionalized cellulose fibers and the unmodified one were recorded within the wavenumber range of 4000–400 cm^−1^ using a PerkinElmer Spectrum 65 with the resolution of 4 cm^−1^. The Malvern Zeta Sizer (ZEN 3600) was used to measure the zeta-potential of the fibers. Microscopic photos of sediments and fungal growth were captured by Nikon Eclipse E200. Elemental analysis of the modified fibers was performed using Thermo Scientific FlashEA 1112.

### Biodegradation study

A qualitative method was used for biodegradability test (Das et al. 2013; Vijan et al. 2012). It is well known that cellulose is susceptible to deterioration by fungi especially *Trichoderma reesei* (Taherzadeh-Ghahfarokhi et al. 2019). Mandel’s agar medium was prepared (Mandels and Weber 1969), and agar surface was cultivated with *Trichoderma reesei* (ATCC 13,631). Subsequently, the cationized fibers were placed on the surface of agar plate to be used as carbon source by the organism. The fibers were examined for colony growth after incubation for 12 days at 28 °C.

### Flocculation experiments

Jar tests were conducted to evaluate efficiency of the cationic fibers to remove colloidal particles from synthetic wastewater. A kaolin suspension with the concentration of 7.5 g/l at pH 7.5 was employed as a stock to prepare working wastewaters at specific turbidities. Then, the pH of the mixture was adjusted to known values using 0.1 M HCl and NaOH. Then, 400 ml of the suspension (working wastewater) was stirred vigorously at 180 rpm for 3 min, followed by adding a certain amount of the flocculant and slow stirring at 40 rpm for 15 min (Nourani et al. 2016). Finally, the suspension was allowed to settle for 45 min, quiescently. During the settling period, samples from supernatant of the wastewater were collected each 15 min to measure turbidity using HACH TURBIDIMETER (Model 2100AN). Each set of experiment had its own blank performed at the similar condition in the absence of flocculant. Then turbidity removal was calculated as follows:

1 $$Turbidity\, removal (\%)= \frac{{T}_{bt}-{T}_{st}}{{T}_{bt}}\times 100,$$

where *T*_*bt*_ and *T*_*st*_ are turbidities of the blank and sample at the same settling time *t*, respectively.

## Results and discussion

### Cellulose fibers

Raw rice husk was milled, treated with NaOH solution and bleached to prepare cellulose fibers. Typically, the procedure yields 5 g bleached fiber per 20 g raw rice husk. The end product slightly kept its natural color when the fibers bleached by the solution of acetic acid and NaOH. However, bleaching the fibers by the solution of acetic acid and aqueous chlorite created white cellulose fibers which were employed in the further studies.

### Preliminary synthesis of the cationized fibers

First, cationization of the cellulose fibers in the presence of pyridine was carried out. The application of prepared fibers in coagulation–flocculation gave an undesirable color to the wastewater due to the use of pyridine in the synthesis reaction. On the other hand, there are potential environmental and health concerns about this catalyst. Therefore, cationization in the absence of catalyst was carried out in subsequent studies.

For this purpose, according to Table 1, various amounts of the reactants were dispersed in 10 ml dimethylformamide and used to synthesize four functionalized fiber samples named P1, P2, P3, and P4. The synthesized products were tested to remove turbidity from the kaolin suspension at pH 6. P1 and P2 did not exhibit the flocculation ability. In contrast, P3 and P4 were able to remove turbidity, the zeta potentials of which were + 8.76 and + 3.97 mV, respectively. Positive zeta-potential values confirmed the effectiveness of the cationization process in the absence of catalyst. In addition, it was found that the zeta-potential values highly depended on the amounts of cellulose and the other chemicals in the reaction environment. Hence, the cationization process was subjected to optimization. It is worth mentioning that triethylamine with basic nature has the potential to act as a catalyst in the reaction, although it is not considered as catalyst in this work.

### Optimizing cationization of the fibers

**Fig. 2:**
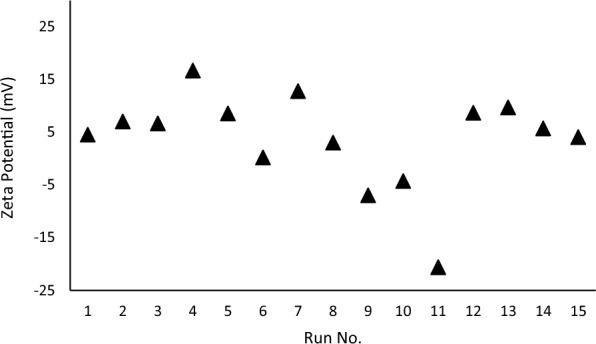
The zeta-potential of the chemically modified fibers obtained in different experiments suggested by Box–Behnken design

The preliminary experiments confirmed that the content of epichlorohydrin, triethylamine, and cellulose in the reaction environment substantially affected the zeta-potential of the modified fibers. Thus, several experiments were carried out in accordance with Box–Behnken design to obtain the fibers with the highest zeta-potential as provided in Table 3. Afterwards, the corresponding products were analyzed at pH 6.5 for zeta-potential. As shown in Fig. 2, zeta-potential of the samples varied from − 20.6 to + 16.7 mV. Then, the results were analyzed to find out the precise model for predicting the zeta-potential from the variables. The corresponding equation is as follows:

2 $${\text{Zeta - potential (mV) }} = \, - 77.15971 - 14.50515A\, + \,20.31354B - 0.357472C\, +\, 0.44300BC\, + \,1.76926A{^2} - 1.21093B{^2} - 0.13223C{^2},$$

where A, B, and C are the amounts of epichlorohydrin (ml), triethylamine (ml), and cellulose suspension (ml), respectively. The ANOVA was carried out on the data set as shown in Table 4. According to *F*-value and *p*-value < 0.05, the equation predicting the responses were significant, while the lack-of-fit values were not significant. Several equation terms including B, BC, A^2^, and C^2^ with *p*-value < 0.05 were significant. Thus, the interaction between B and C affecting the response was also proved. Interaction between variables occurs whenever the effect of one variable is dependent upon the level of another one.

**Table 4:** Analysis of variance table for the model predicting zeta-potential of the chemically modified fibers

Source	Sum of squares	df	Mean square	*F* value	*p*-value Prob > *F*
Model	934.08	7	133.44	4.51	0.0326
A-Epichlorohydrin	3.94	1	3.94	0.1332	0.7259
B-Triethylamine	240.24	1	240.24	8.11	0.0248
C-Cellulose suspension	5.02	1	5.02	0.1695	0.6929
BC	176.62	1	176.62	5.96	0.0446
A^2^	184.93	1	184.93	6.24	0.0411
B^2^	86.63	1	86.63	2.93	0.131
C^2^	204.27	1	204.27	6.9	0.0341
Residual	207.31	7	29.62		
Lack of fit	190.55	5	38.11	4.55	0.19
Pure error	16.75	2	8.38		
Cor total	1141.39	14			

To validate the experimental design, two flocculant samples were prepared at an optimal condition (suggested by the software) using 6.0 ml epichlorohydrin, 11.74 ml triethylamine, and 18.3 ml cellulose suspension (1 g fibers/15 ml DMF). The zeta-potential of synthesized fibers was 15.2 ± 1.0 mV which was consistent with the predicted value of 15.4 ± 3.63 mV. Compared with the preliminary experiments, an increment of 73% in zeta-potential was obtained using the optimization method. This sample was used for further studies, unless specified otherwise.

### Characteristics of the fibers

The chemically modified fibers were introduced to the following characterizations.

#### FTIR and elemental analyses

**Fig. 3:**
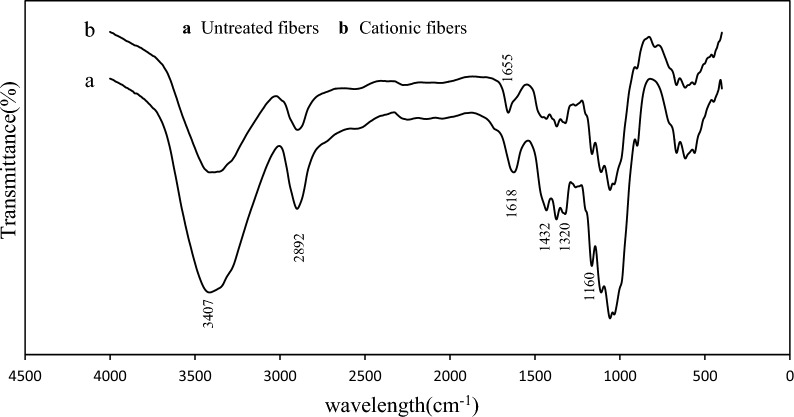
FTIR analysis for the untreated fibers and cationic one

Raw and modified cellulose fibers were subjected to FTIR analysis in the range of 400 to 4000 cm^−1^ to recognize the corresponding functional groups on their structure. As shown in Fig. 3a, b, several absorption peaks at 3407 cm^−1^ (–OH stretching bonds), 2892 cm^−1^ (symmetric C–H stretching vibration), 1432 cm^−1^ (asymmetric angular deformation of C–H), 1320 cm^−1^ (CH_2_ wagging), and 1160 cm^−1^ (C–O–C stretching bonds) were observed (Zhuang et al. 2020; Hospodarova et al. 2018). The band at 1618 cm^−1^ was due to O–H bending in absorbed water, which showed a shift to 1655 cm^−1^ after cationization (Aguado et al. 2017).

Moreover, two samples (Runs # 4 and 11 in Table 3) with zeta potentials of + 16.7 and -20.6 mV were subjected to elemental analysis. These samples (Runs # 4 and 11) contained N (0.63% and 0.85%), H (6.45% and 6.89%), and C (43.34% and 44.18%), respectively. The presence of N in the samples confirmed the occurrence of the reaction between triethylamine and epichlorohydrin-treated fibers. Positive surface charges in addition to elemental analysis proved the formation of quaternary amine in the structure of the synthesized samples. Cellulose fibers bear negative surface charges. Thus, the considerable amount of positively charged groups should be grafted to the fiber to obtain net positive charge. The chemical modification at non-optimal reactant-to-fiber ratios as well as low concentration of reactants led to the formation of quaternary amine at insufficient amounts on the fibers. Consequently, the net negative surface charge was observed for some samples. The optimal condition noted in the validation experiment was in consistence with this explanation.

#### Zeta-potential at different pHs

**Fig. 4:**
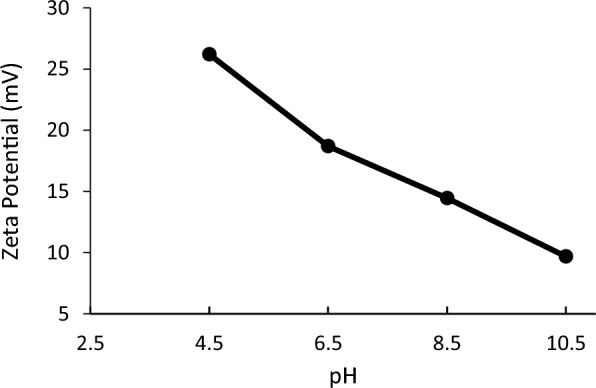
Variation of zeta-potential of the modified fibers suspension (1 g/l) against pH. The surface charges of the synthesized fibers remained positive in the pH range

The fibers synthesized at the optimal condition were used to study variation of surface charge with pH. To this end, the suspensions of the modified fibers (1 g/l) at various pHs between 4.5 and 10.5 were prepared. The surface charge of the samples was measured. The cationic property of the modified fibers at such a pH range was proved, which was attributed to the presence of quaternary ammonium on the surface of cellulose fibers (Fig. 4).

In a previous study, cationic cellulose was prepared using 3‐chloro‐2‐hydroxypropyl‐trimethylammonium chloride. The modified cellulose had cationic properties at different pHs. However, the zeta-potential values of the prepared samples were approximately lower than those found in the present study. The synthesized product was subsequently grafted with polyacrylamide. The zeta-potential of the grafted cellulose was pH-dependent with positive surface charge only at acidic condition (Zhang et al. 2016). However, the flocculant with higher zeta-potential can be obtained by applying pyridine as a catalyst in the synthesis reaction (Gao et al. 2009). In another study, epichlorohydrin reacted with ammonium hydroxide to produce 2-hydroxy-3-chloro propyl amine. The reaction product was used to graft cellulose nanocrystals through the etherification reaction in an organic environment. The functionalized cellulose nanocrystals showed the positive surface charge at the pH lower than 8 (Akhlaghi et al. 2015). As an advantage, the modified fibers developed in this study had absolutely positive surface charges at a broad pH range.

### Biodegradability of the flocculant

Certain microorganisms are capable to convert the carbon present in cellulosic matters into carbon dioxide, biomass and other products through metabolic processes. This is mainly achievable using those microorganisms that produce cellulase enzyme, e.g., *Trichoderma reesi* (Taherzadeh-Ghahfarokhi et al. 2021). This microorganism can secrete cellulases which hydrolyze the biopolymer chains into short fragments for metabolization. The fungal growth on the medium containing the modified fibers as a sole carbon source was observed (Additional file 1: Fig. S1), which proved the consumption and hydrolysis of the flocculant. Thus, the modified fibers were biodegradable (Das et al. 2013; Vijan et al. 2012).

### Flocculation efficiency

In previous studies, cationized cellulose fibers originated from other agricultural sources have frequently been applied for removing dissolved solids. However, wastewater often includes suspended and colloidal particles besides dissolved ones. Hence, the ability of cationic fibers to remove colloidal particles was examined via coagulation–flocculation process. To this end, kaolin suspensions with various initial turbidities (20, 200 and 500 NTU correspond to 0.03, 0.21 and 0.54 g/l, respectively) at different pHs (6, 7, and 8) were prepared and used as synthetic wastewater. Jar tests were performed by adding several dosages of flocculant (25, 50, 100, 150, 200, 400, 500 mg/l) to the wastewater, and the suspension turbidity was measured every 15 min.

#### Initial turbidity of 500 NTU

**Fig. 5:**
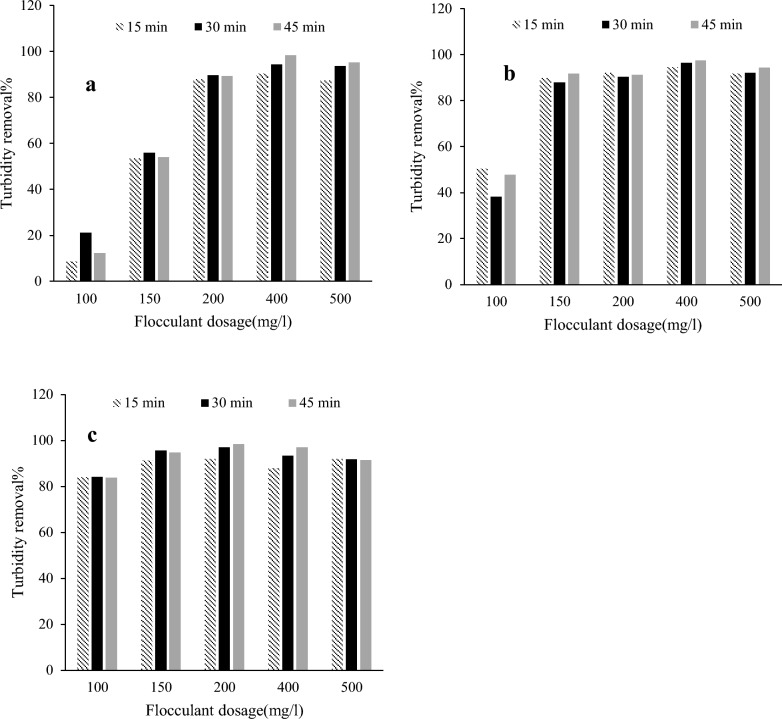
The performance of the synthesized flocculant for turbidity removal from kaolin suspensions at the initial turbidity of 500 NTU considering different settling times at **a** pH 6, **b** pH 7, and **c** pH 8

At pH 6, applying the flocculant at the dosage of 200 mg/l, resulted in a fast turbidity removal of about 88% after 15-min settling while the turbidity removal did not substantially change by passing the time (Fig. 5). At lower dosages of 100 and 150 mg/l, turbidity removals up to 56% were observed considering the fact that sediment became partially re-suspended by passing the time after 30 min. In other words, the formed flocs were not stable and tight by using the lowest dosage of flocculant (100 mg/l). However, treating the wastewater with the dose of 400 mg/l created a turbidity removal higher than 98% after 45-min settling, while increasing the flocculant to 500 mg/l led to a minor decrease in turbidity removal (95%). The same experiments were conducted using the untreated cellulose fibers. The resulting turbidity removals were negative for all doses of the untreated fibers after 30-min settling. In other words, the untreated fibers bearing negative charge were not able to improve turbidity removal.

Using 150 mg/l flocculant at pH 7 caused a fast turbidity removal about 90% after 15-min settling while the turbidity removal was approximately constant by passing the time. Similar behavior was approximately observed where 200 mg/l flocculant was employed to treat the wastewater. At the lower dosages of 100 mg/l, slow turbidity removal about 50% was recorded after settling for 15 min while the sediment was re-suspended by passing the time as observed at pH 6. However, applying 400 mg/l flocculant to the experiments caused a turbidity removal of 97.5% after 45-min settling, while increasing the flocculant concentration to 500 mg/l decreased the turbidity removal percentage.

At pH 8, applying 100 mg/l flocculant caused a fast turbidity removal of about 84% after 15-min settling while the turbidity removal was constant by passing the time. Treating the wastewater with the higher doses of flocculant (150, 200 and 400 mg/l) caused a gradual increase in turbidity removal between 95 and 98.5%. Using such flocculant dosages, the settling was observed slower so that it needed 30 min for the dosage of 150 mg/l, and 45 min for 200 and 400 mg/l. Increasing the flocculant to 500 mg/l decreased the turbidity removal as observed for the other pHs.

The results proved that interesting turbidity removals up to 98.5% could be obtained where the initial turbidity of the synthetic wastewater was 500 NTU. Varying the pH of wastewater did not substantially affect turbidity removals if the high flocculant doses (200, 400 and 500 mg/l) were applied. In addition, the lowest dosage of 100 and 150 mg/l displayed the better performance at the higher pHs.

#### Initial turbidity of 200 NTU

**Fig. 6:**
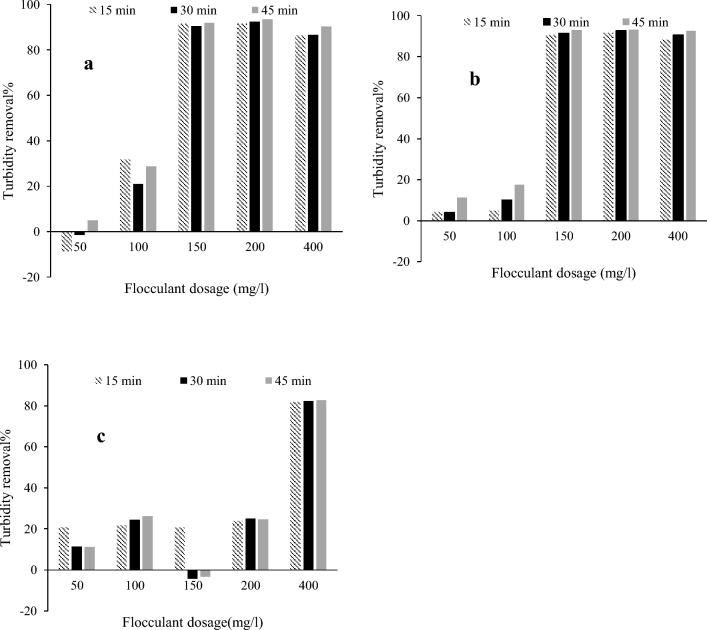
The performance of the synthesized flocculant for turbidity removal from kaolin suspensions at the initial turbidity of 200 NTU considering different settling times at **a** pH 6, **b** pH 7, and **c** pH 8

The turbidity removal was investigated by adding the flocculant to the synthetic wastewater with initial turbidity of 200 NTU (Fig. 6). At pH 6, the turbidity removals higher than 91% were achieved using 150 and 200 mg/l flocculant after 15-min settling. It reached to more than 93% removal after 45-min settling with 200 mg/l flocculant. The higher flocculant dosage (400 mg/l) was not more effective. The lowest flocculant (50 mg/l) did not show the turbidity removal efficiency; particularly it increased the turbidity at the first 30-min settling. Low portion of the turbidity was removed using 100 mg/l cationic fibers.

At pH 7, 50 and 100 mg/l flocculant offered turbidity removals lower than 20%. In contrast, the turbidity removals better than 88% were observed using the higher flocculant dosages after only 15-min settling. Turbidity removals reached about 93% after 45-min settling with 200 mg/l flocculant. For the wastewater treatment at pH 8, using 400 mg/l flocculant caused a turbidity removal about 83%. However, the lower dosages were able to remove the turbidity up to 26%. For the flocculation experiments using the 200 NTU-turbid wastewater, the high removals between 83 and 93% could be obtained at the different pHs.

#### Initial turbidity of 20 NTU

The flocculation experiments were carried out using the wastewater with initial turbidity of 20 NTU (data are not shown). For the experiment at pH 6, the turbidity removal of 30% was observed with 100 mg/l flocculant after 15-min settling and the removal was decreased by increasing the settling times. The lower and higher dosages of flocculant displayed the lower turbidity removals whereas the turbidity was higher than that of the blank experiment after 45-min settling. In other words, the flocs formed by adding the flocculant were unstable and re-suspended after settlement. At pH 7, the turbidity removal of 25% was obtained using 100 mg/l flocculant after 15-min settling while the other dosages and settling times showed the lower removals. In most experiments at pH 7, the turbidity removal decreased by increasing the settling times, supporting instability of the formed flocs. Compared to the blank sample, the turbidity of the wastewater increased after 15-min settling using 150 mg/l flocculant. The jar tests at pH 8 resulted in the turbidity removals up to 20%. The settling was slow such a way that the better turbidity removals were reached after 30 min for all applied flocculant doses. The formed flocs were also unstable at this pH. Using the wastewater with the turbidity of 20 NTU, the turbidity removals between 20 and 30% could be obtained considering the fact that the removals slightly decreased with increment of pH.

In accordance with our findings, it is reported that increasing the flocculant dosage destabilizes colloidal suspension, consequently agglomeration of kaolin particles and formation of larger flocs initiates. However higher dosages of flocculant may cause cage effect and prevent the flocs to become larger and stronger. As a result, efficiency of sedimentation decreases (Ma et al. 2017). Moreover, effluent color was not affected by applying this flocculant to wastewater treatment while the fibers cationized in the presence of pyridine led to new challenge by giving unwanted color to the wastewater. The change in effluent color obligates excess treatment of wastewater streams.

### Comparison of the results

In order to provide comparison between the synthetic flocculant and conventional ones, PACl was used to remove turbidity from the same suspension (Additional file 1: Fig. S2). At pH 6, the highest turbidity removal after 45-min settling was lower than 18% by applying 3 mg/l PACl. However, the excellent turbidity removal of about 88% was found using 200 mg/l cationic fibers after 15-min settling. At pH 7, adding 0.25 mg/l PACl to the wastewater resulted in about 90% removal after 15 min, while the removals higher than 94% and 97% were obtained using 400 mg/l fibers after 15 and 45-min settling, respectively. At pH 8, the highest turbidity removal was referred to 0.25 mg/l of PACl, displaying about 84% turbidity reduction after 15 min. At the same condition, the synthetic flocculant showed 98.5% turbidity removal at 200 mg/l dosage. It can be generally concluded that, significantly higher removal can be obtained using the developed flocculant at the mentioned conditions. Furthermore, the better results can be probably obtained by optimization of rapid and slow mixing steps (Farasat et al. 2017). Compared with PACl, higher amount of cellulosic flocculant was consumed to obtain such a removal. As a result, higher sludge will be produced using this flocculant in practical applications. However, it should be noted that the developed flocculant was biodegradable and its sludge could be simply managed. In contrast, PACl brings environmental and health issues due to Al remaining in the treated wastewater and the corresponding sludge, even for consumption at lower dosages.

The use of cationic cellulose for turbidity removal from a kaolin suspension (0.20 wt. %) was reported. The synthesized flocculant showing positive zeta potentials in a broad range of pHs (2–12) offered a turbidity removal lower than 90% at best. Furthermore, the turbidity removal was tightly depended on the pH of the suspension, particularly pH values near 7 (Zhang et al. 2016), which was in contrast to our findings. In another study, cationic cellulose samples were synthesized and the corresponding flocculation efficiency was evaluated using a kaolin suspension (0.25 wt. %). At the end of the settling period, the transmittance of the suspension was measured at a wavelength of 420 nm, considering the fact that higher transmittance was equivalent to superior flocculation performance. The maximum transmittance of 80% was obtained by applying the flocculant at pH 7 (Yan et al. 2009). The flocculation performance of another cationic cellulose was evaluated using a kaolin suspension (1%) and determining its residual transmission after analytical centrifugation. The maximum transmission was lower than 60% (Liimatainen et al. 2011). Dialdehyde cellulose was produced by periodate oxidation of birch cellulose pulp, followed by a reaction with (2-hydrazinyl-2-oxoethyl)-trimethylazanium chloride to synthesize a cationic dialdehyde cellulose. The performance of the flocculant was evaluated using ground calcium carbonate suspension (1%) and determining its residual transmission after centrifugation. The maximum transmission was lower than 65% (Sirviö et al. 2011). In the current study the excellent turbidity removal of 98.5% was obtained.

### Flocculation mechanism

**Fig. 7:**
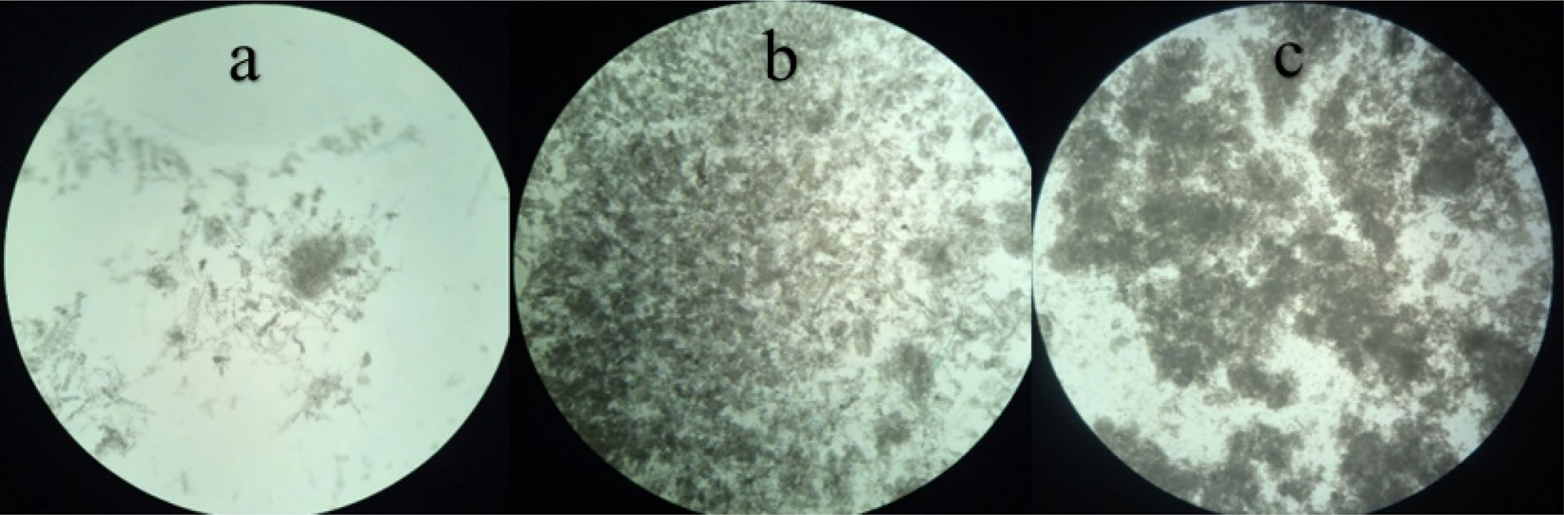
The photography of the flocs formed by adding the cationic cellulose-based flocculant to the wastewater. The initial turbidities of **a** 20 NTU **b** 200 NTU, and **c** 500 NTU

Whereas surface charge of kaolin particles is negative (in pH range 6–8), the corresponding suspension is stable until charge neutralization mechanism occurs. Charge neutralization by adsorbing the opposite charged particles destabilizes the suspension, hence the settlement is observed. On the other hand, the polymeric nature of cellulosic flocculants suggests bridging as one of the main governing mechanisms (Li et al. 2018). In this study, the synthesized flocculant carrying absolutely positive surface charge was applied in the absence of traditional coagulants. Thus both charge neutralization and bridging were governing mechanisms for flocculation. However, the dominant mechanism was adsorptive charge neutralization where the diluted wastewater (the initial turbidity of 20 NTU) was used, since the flocs were small (Fig. 7). The other researchers have also reported that charge neutralization gives a smaller floc size compared to bridging flocculation (Liimatainen et al. 2011). Moreover, the largest flocs observed in the experiments using the most turbid wastewater (500 NTU) confirmed the presence of bridging as main mechanism. The mid-size flocs appeared in the experiments with the initial turbidity of 200 NTU supported the governance of both charge neutralization and bridging mechanisms.

## Conclusions

Rice husk, a lignocellulosic agricultural waste, was managed and valorized by converting it to cationic fibers. Pyridine is regularly used as a catalyst for cationization of cellulose fibers. To address the environmental concern connect with this catalyst, cationization of the fibers was successfully performed and optimized in the absence of catalyst. In previous studies, cationized cellulose fibers originated from other agricultural sources have frequently been applied for removing dissolved solids. However, wastewater often includes suspended and colloidal particles besides dissolved ones. Hence, the synthesized cationic fibers were used as flocculant to remove colloidal particles from the synthetic wastewater. As a result, excellent turbidity removals up to 98.5% were obtained, which was higher than those reported in the literatures, to the best of our knowledge. For initial turbidity of 500 NTU, the flocculation performance of the modified fibers was not affected by variation of the wastewater pH. In contrast to catalytically cationized fibers, the synthesized flocculant did not affect the effluent color. The governing mechanisms of flocculation were bridging and charge neutralization through adsorption, considering the fact that initial turgidities of the wastewater affected the dominant mechanism. Substantial advantages such as high performance for turbidity removal and suitable operational characteristics are associated with the use of this flocculant. Thus, the procedure can be applied on lignocellulosic wastes to develop cationic fiber with effective flocculation ability.

### Supplementary Information

**Additional file 1: Fig. S1** Microscope picture of *T. reesei* growth on the cationized fibers (100 × magnification). **Fig. S2** The PACl performance for turbidity removal from kaolin suspensions at the initial turbidity of 500 NTU considering different settling times at **a** pH 6, **b** pH 7, and **c** pH 8.

## Data Availability

All the needed data are provided in the manuscript.

## Abbreviations

DMF: Dimethylformamide
RSM: Response surface methodology
FTIR: Fourier transform infrared
