# Supplementary material for: Conversion of lignocellulosic waste into effective flocculants: synthesis, characterization, and performance
Source: Bioresour Bioprocess. 2021 Aug 3;8(1):69. doi: 10.1186/s40643-021-00422-1 (PMC11341801; doi:10.1186/s40643-021-00422-1)
Supplement: Supplementary file 1 — Additional file 1: Fig. S1 Microscope picture of T. reesei growth on the cationized fibers (100 × magnification). Fig. S2 The PACl performance for turbidity removal from kaolin suspensions at the initial turbidity of 500 NTU considering different settling times at a pH 6, b pH 7, and c pH 8. [file 40643_2021_422_MOESM1_ESM.docx]

**Supporting Information**

**Conversion of lignocellulosic waste into effective flocculants: Synthesis, characterization, and performance**

Elham Jahedi, Reza Panahi*

Chemistry & Chemical Engineering Research Center of Iran (CCERCI), Tehran, Iran

*Corresponding author.

Tel. /Fax: +98 21 44787819.

*E-mail address:* Panahi@ccerci.ac.ir (R. Panahi).


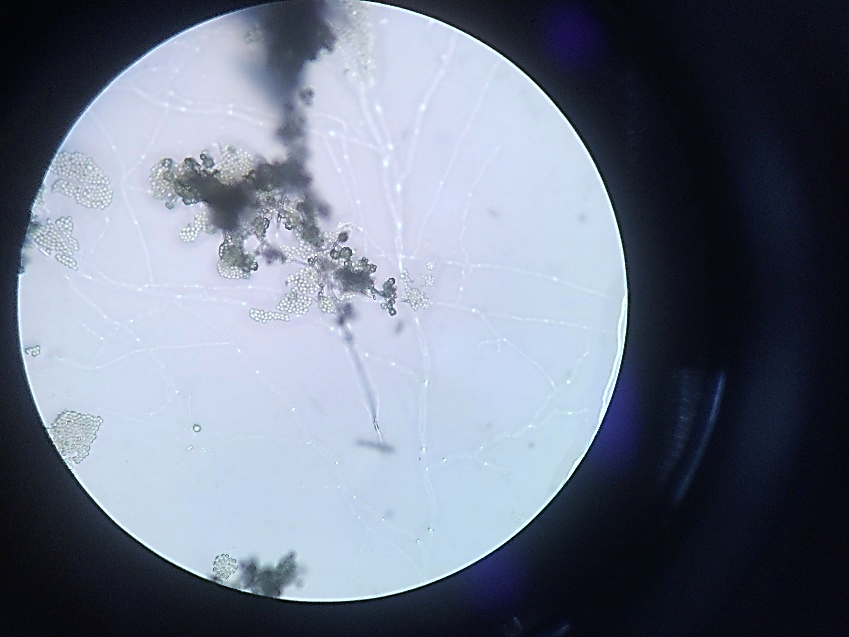


**Fig. S1** Microscope picture of *T. reesei* growth on the cationized fibers (100 × magnification).

**Fig. S2** The PACl performance for turbidity removal from kaolin suspensions at the initial turbidity of 500 NTU considering different settling times at (a) pH 6, (b) pH 7, and (c) pH 8
